# Neuromuscular sonography detects changes in muscle echotexture and nerve diameter in ICU patients within 24 h

**DOI:** 10.1007/s40477-021-00621-8

**Published:** 2021-12-06

**Authors:** Catherine Bulinski, Maxime Viard, Alexander Vlazak, Kathrin Habig, Martin Juenemann, Christoph Best, Ingo Schirotzek, Manfred Kaps, Heidrun H. Krämer

**Affiliations:** 1 Department of Neurology, Justus Liebig University, Klinikstrasse 33, 35392 Giessen, Germany; 2 Department of Neurology, Phillipps University, Baldingerstrasse 1, Marburg, Germany

**Keywords:** ICU-AW, Muscle ultrasound, Nerve ultrasound, Gray-scale analysis, Cross-sectional area

## Abstract

**Purpose:**

During an ICU stay, changes in muscles and nerves occur that is accessible via neuromuscular sonography.

**Methods:**

17 patients recruited from the neurological and neurosurgical ICU (six women; 66 ± 3 years) and 7 healthy controls (three women, 75 ± 3 years) were included. Muscle sonography (rectus abdominis, biceps, rectus femoris and tibialis anterior muscles) using gray-scale values (GSVs), and nerve ultrasound (peroneal, tibial and sural nerves) analyzing the cross-sectional area (CSA) were performed on days 1 (t1), 3 (t2), 5 (t3), 8 (t4), and 16 (t5) after admission.

**Results:**

Time course analysis revealed that GSVs were significantly higher within the patient group for all of the investigated muscles (rectus abdominis: *F* = 7.536; *p* = 0.011; biceps: *F* = 14.761; *p* = 0.001; rectus femoris: *F* = 9.455; *p* = 0.005; tibialis anterior: *F* = 7.282; *p* = 0.012). The higher GSVs were already visible at t1 or, at the latest, at t2 (tibialis anterior muscles). CSA was enlarged in all of the investigated nerves in the patient group (peroneal nerve: *F* = 7.129; *p* = 0.014; tibial nerve: *F* = 28.976, *p* < 0.001; sural nerve: *F* = 13.051; *p* = 0.001). The changes were visible very early (tibial nerve: t1; peroneal nerve: t2). The CSA of the motor nerves showed an association with the ventilation time and days within the ICU (t1 through t4; *p* < 0.05).

**Discussion:**

We detected very early changes in the muscles and nerves of ICU-patients. Nerve CSA might be a useful parameter to identify patients who are at risk for difficult weaning. Therefore our observations might be severity signs of neuromuscular suffering for the most severe patients.

## Introduction

Depending on the patient collective, about 46% of patients requiring treatment in the intensive care unit (ICU) develop neuromuscular weakness (intensive care unit acquired weakness, ICU-AW) [1]. Critical illness myopathy (CIM), critical illness polyneuropathy (CIP), and the combination of the two are the most common causes of the failure to wean from the ventilator as well as ICU-AW [2]. The timely identification of individuals who are at risk of developing severe ICU-AW is crucial yet challenging because the presence of CIM and CIP defines the patient’s clinical outcome and his or her quality of life after being discharged from the hospital [3].

The sequence of events initiating CIM and CIP is complicated and still under investigation. CIM is defined as an acute primary myopathy. The clinical hallmark is severe muscular atrophy [4]. In biopsy [5] and motor and sensory nerve conduction studies [6, 7], CIP is an acute sensory-motor axonal polyneuropathy. Among other mechanisms, the disturbance of microcirculation; inflammatory processes [8]; metabolic alterations, such as hyperglycaemia; mitochondrial failure; catabolic pathway activation; and sodium channel dysfunction [2] are part of the pathophysiology. Moreover, CIM leads to structural and functional muscle changes due to reduced synthesis and the increased consumption of proteins (e.g., myosin) [4].

Diagnostic of CIM and CIP relies on clinical and neurophysiological examination. The diagnostic value of nerve and muscle sonography in the diagnosis of ICU-AW has been subject of different studies. Kelmenson and colleagues found a sensitivity of 82% and a specificity of 57% of muscle ultrasound for the diagnosis of CIM and CIP if the echogenicity was increased [7]. Witteveen and colleagues found quantitative muscle ultrasound to be insufficient for discriminating between patients with and without ICU-AW upon awakening [9]. In this previous study, nerve sonography was unable to distinguish between patients with and without ICU-AW upon awakening [9].

In the present study, we aimed to investigate changes in muscle echogenicity via gray-scale analysis (gray-scale values; GSVs) and changes in the cross-sectional area (CSA) of nerves in a mixed collective of neurological and neurosurgical ICU patients within a period of 16 days after admission.

## Materials and methods

### Participants

17 patients (six women; mean age 66 ± 3 years) and seven healthy controls (three women, mean age 75 ± 3 years) were included. The patients were recruited from the ICUs of the Department of Neurology and Neurosurgery, Justus-Liebig-University, Giessen. The inclusion criteria included an expected ventilation time and an ICU stay of ≥ 7 days. Patients were excluded if they had amputations, had primary neuromuscular pathologies, or were extubated and left the ICU earlier than 7 days after admission.

Informed and written consent according to the Declaration of Helsinki from a legal guardian or a close relative was obtained upon inclusion, with retrospective patient consent obtained later.

The healthy age- and sex-matched participants were drawn from the staff and their relatives of the Department of Neurology, Justus-Liebig-University, Giessen. Written consent was obtained from the healthy controls.

The study received approval from the local ethics committee of the medical association of the Justus-Liebig-University, Giessen (182/14).

The patients were investigated within 24 h after admittance to the ICU (t1) and after three (t2), five (t3), eight (t4), and 16 (t5) days. The healthy participants underwent the same study protocol.

## Neuromuscular ultrasound

Measurements were carried out using a Phillips EPIQ7 with a 5–18 MHz linear array transducer (Philips Medical Systems, Bothell, WA). The same investigator (CB) performed high-resolution ultrasound scans in B-mode bilaterally.

### Muscle ultrasonography

**Fig. 1:**
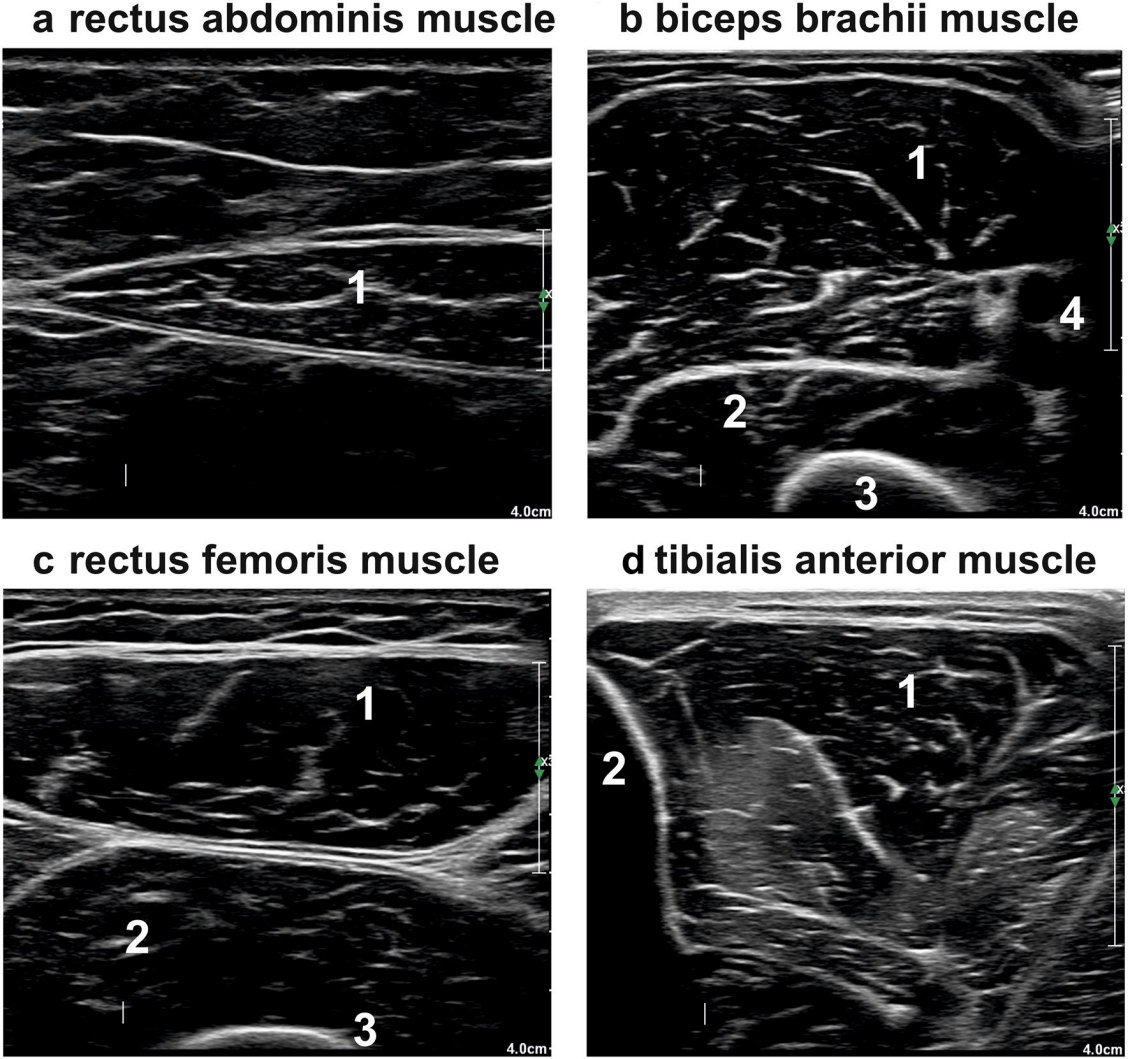
Depicts examples of sectional sonographic images of muscles surrounded by the typical anatomic landmarks **a**: rectus abdominis muscle (1: rectus abdominis muscle); **b**: biceps brachii muscle (1: biceps brachii muscle; 2: brachial muscle; 3: humerus; 4: neurovascular bundle); **c**: rectus femoris muscle (1: rectus femoris; 2: vastus intermedius muscle; 3: femur); **d**: tibialis anterior muscle: (1: tibialis anterior muscle; 2: tibia).

The participants were investigated while resting in a supine position. A generous amount of contact gel was used to minimize the required pressure of the transducer that was placed vertically on the skin. The equipment setting were identical throughout all measurements (gain: 48%; dynamic range: 68 dB; monitor brightness: 51%). Transverse ultrasound scans were made of the following muscles bilaterally using a standardized protocol described previously (transducer positions include biceps muscles: two-thirds of the distance from the acromion to the antecubital crease; rectus abdominis muscles: 2 cm cranially from the umbilicus [10]; rectus femoris muscles: half of the distance from the anterior–superior iliac spine and the superior aspect of the patellae; and tibialis anterior muscles: one-quarter of the distance between the inferior aspect of the patella and the lateral malleolus [10, 11]; for details see Fig. 1). For the calculation of echogenicity, three images were taken in each investigated section to minimize the measurement variability.

The mean echogenicity was analyzed offline using gray-scale analysis as previously described [12]. In each of the three images from each muscle, the region of interest (ROI) was selected within the borders of the surrounding structures. The mean echogenicity of the ROI was calculated using a standard histogram function (Corel Photo-Paint (X6); eight-bit resolution resulting in a number between 0 and 255, where black = 0, white = 255) and averaged over the three measurements per muscle. The mean of the gray-scale value (GSV) from all three acquired pictures was used for further analysis [13].

### Nerve sonography

The nerves were identified on transverse scans using anatomical landmarks. Healthy subjects were positioned on the side with the knee slightly flexed. In brief, the peroneal nerve was examined at the level of the fibular neck [14]. The tibial nerve was assessed in the popliteal fossa anterior to the popliteal artery [15]. The sural nerve was obtained above the lateral malleolus [16].

**Fig. 2:**
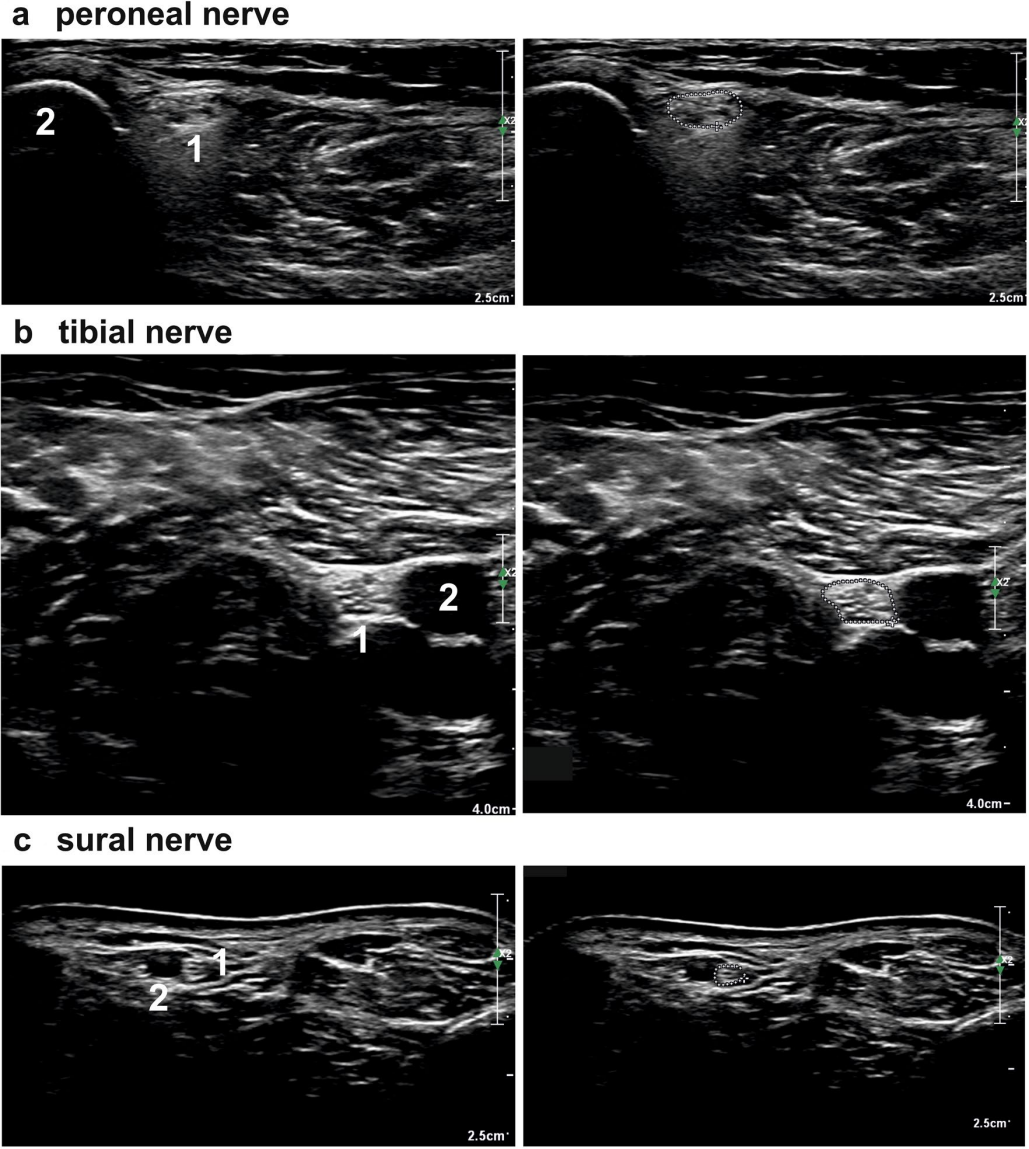
Depicts examples of sectional sonographic images of nerves surrounded by the typical anatomic landmarks. There are two sections provided for each nerve, with and without the marking of the cross sectional area (**a**: peroneal nerve (1: peroneal nerve; 2: fibula); **b**: tibial nerve (1: tibial nerve; 2: arteria poplitea); **c**: sural nerve (1: sural nerve; 2: small saphenous vein).

The cross-sectional area (CSA) was measured with the trace function along the inner hyperechoic rim on transverse sonograms. As for the muscles, each nerve was measured three times, and the values were averaged. For details see Fig. 2.

### Compound muscle action potential (CMAP)

In the patient group, the CMAP of both tibial nerves was obtained at all designated investigation points (Nicolet Viking; Natus Medical, Middleton, WI, USA). In the control group, the CMAP was acquired only once. In brief, the active electrode was put over the abductor halluces and the reference electrode on the metatarsophalangeal junction of the first toe. The stimulation occurred at the medial ankle [17].

### Laboratory tests

For the purpose of identifying associations between laboratory parameters and muscle and nerve changes, the following parameters were acquired in the investigated groups at all investigation points: cholesterol including high-density lipoproteins and low-density lipoproteins, triglycerides, lipoprotein(a), interleukin 6, C-peptide, HbA1c, glucose, creatine kinase, lactate, pyruvate, and procalcitonin.

### Statistics

Data were analyzed using the SPSS Statistics (IBM, Version 23.0 for Windows) software package. We calculated separate analyses of variance for GSVs (muscle sonography), CSA (nerve sonography), and CMAP (motor conduction studies) with “patients” and “healthy controls” as main factors. Subgroup comparisons of the main factors were performed via post-hoc tests. Greenhouse–Geisser epsilon was used to correct for the violation of sphericity. For the purpose of elucidating a connection among clinical data, laboratory values, and sonographic parameters, as well as CMAP, Pearson or respective Spearman correlation tests were calculated. Kolmogorov–Smirnov tests of normality were run for all data sets, and parametric (*t* tests) or non-parametric statistics (Mann–Whitney test) were used accordingly. All values were given as means ± standard error (SEM) in the case of a normal distribution, as well as the medians and IQR in the case of a non-normal distribution. Values were considered to be significant if *p* < 0.05.

## Results

### Patients

From 25 recruited patients, only 17 patients completed the study protocol. Reasons for dropout include extubation after fewer than 7 days of ventilation (*n* = 3), transfer to another ward (*n* = 3), the withdrawal of consent to participate (*n* = 1), and death (*n* = 1). Two patients died 10 and 12 days after admission and therefore missed t5 (t5: *n* = 15; t1–t4: *n* = 17).

Patients stayed in the ICU for 26 days (median; IQR: 21.0–34.0). The invasive ventilation time was 20 days (median; IQR: 10.0–31.5).

A positive correlation between the time on the artificial respirator and the duration of the ICU stay (*p* < 0.001; *r* = 0.803; Spearman) was found. Patients who did not survive stayed longer in the ICU (*p* = 0.004; *r* = 0.666).

Clinical and biographical data are depicted in Table 1.

**Table 1:** Biographical and clinical patient data

Patient number	Age (y)	Sex	Admission diagnosis	Invasive ventilation (d)	ICU LOS (d)	Mean fluid balance (24 h in ml over 7 d)
1	55	M	Right-sided intracerebral hemorrhage with intraventricular extension	49	49	790.00 ± 361.92
2	71	M	Status epilepticus	44	44	795.00 ± 215.65
3	79	M	Left-sided intracerebral hemorrhage from ruptured aneurysm	139	140	1122.29 ± 726.75
4	63	M	Left-sided intracerebral hemorrhage with intraventicular extension	7	17	332.43 ± 414.86
5	73	F	Bacterial meningitis caused stemming from streptococcus pneumoniae	9	22	655.93 ± 316.31
6	43	M	Traumatic brain injury	26	26	-21.57 ± 442.26
7	59	M	Thrombosis of the BA	20	31	412.76 ± 236.59
8	71	F	Right-sided intracerebral hemorrhage with intraventricular extension	36	36	497.14 ± 348.38
9	65	M	Right-sided intracerebral hemorrhage with intraventricular extension	25	25	1160.43 ± 591.35
10	79	M	Traumatic left-sided subdural hematoma	18	22	867.00 ± 158.49
11	73	F	Right-sided intracerebral hemorrhage with midline shift	7	23	794.29 ± 113.54
12	69	F	Cerebral ischemia by occlusion of the left MCA	12	20	993.71 ± 473.28
13	53	M	Cerebral ischemia by occlusion of the right MCA	26	26	380.57 ± 320.26
14	83	F	Cerebral ischemia by occlusion of the left CCA	8	10	1455.43 ± 335.76
15	78	M	right-sided intracranial hemorrhage with intraventricular extension	12	12	253.09 ± 313.16
16	70	M	Right-sided intracranial hemorrhage with intraventricular extension	11	32	682.06 ± 217.78
17	42	F	Cerebral ischemia by occlusion of the right MCA	27	27	739.86 ± 92.60

Comorbidities
Arterial hypertension *n* = 17
Coronary heart disease *n* = 3
Chronic obstructive pulmonary disease *n* = 2
Asthma bronchiale *n* = 1
Occlusive peripheral arterial disease *n* = 1
Melanoma malignum *n* = 1
Epilepsy *n* = 1
Post-traumatic stress disorder *n* = 1
Psoriasis *n* = 1
Glaucoma *n* = 1
Diabetes *n* = 4
Bronchial carcinoma *n* = 1

ICU-associated comorbidities
Pneumonia *n* = 11
Ventriculits: *n* = 2
Urinary tract infection *n* = 5
Renal failure *n* = 2

The mean fluid balance was calculated for 24 h for the first 7 days after ICU admission and does not include the loss of fluids via respiration or sweating

*y* years, *LOS* length of stay, *d* days, *f* female, *m* male, *MCA* middle cerebral artery, *BA* basilar artery, *CCA* common carotid artery

### Muscle sonography

**Fig. 3:**
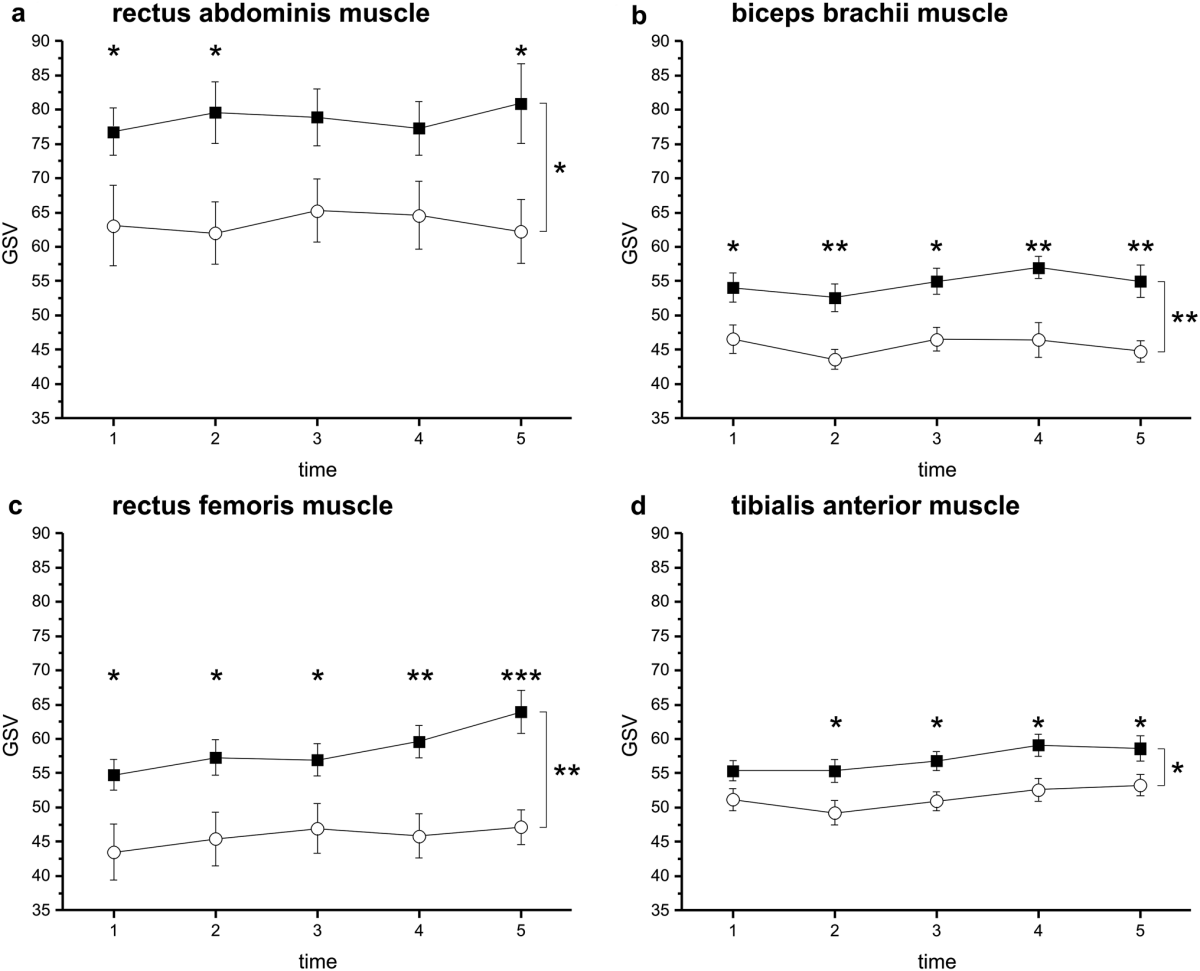
Depicts the time course of gray-scale values (GSV) in ICU patients and the healthy control group. The GSVs are higher in the patients compared with the healthy controls for all investigated muscles (**a**: rectus abdominis: *F* = 7.536; *p* = 0.011; **b**: biceps muscles: *F* = 14.761; *p* = 0.001; **c**: rectus femoris muscles: *F* = 9.455; *p* = 0.005; **d**: tibialis anterior muscles: *F* = 7.282; *p* = 0.012). Statistical details are presented in Table 1. ***p < 0.001; **p < 0.01; *p < 0.05.

Time course analysis detected significantly higher GSVs within the patient group in the rectus abdominis muscles (*F* = 7.536; *p* = 0.011). Post-hoc tests revealed significantly elevated GSVs within the patient group at t1 (*p* = 0.042), t2 (*p* = 0.018) and t5 (*p* = 0.020; *t* tests). For details, see Fig. 3a and Table 2.

**Table 2:** GSV of muscles

Muscles	GSV					
	t1	t2	t3	t4	t5	
Rectus abdominis muscles						
Patients	76.78 ± 3.49	79.59 ± 4.51	78.89 ± 4.14	77.28 ± 3.89	80.89 ± 5.81	
Controls	63.09 ± 5.85	61.98 ± 4.53	65.25 ± 4.61	64.56 ± 4.95	62.20 ± 4.66	
*p*	0.042	0.018	ns	ns	0.020	
Biceps brachii muscles						
Patients	54.06 ± 2.16	52.59 ± 2.02	54.96 ± 1.89	56.97 ± 1.59	54.96 ± 2.35	
Controls	46.58 ± 2.06	43.60 ± 1.42	46.53 ± 1.75	46.45 ± 2.51	44.78 ± 1.53	
* p*	0.017	0.008	0.010	0.001	0.001	
Rectus femoris muscles						
Patients	54.74 ± 2.25	57.27 ± 2.61	56.93 ± 2.39	59.62 ± 2.33	63.92 ± 3.07	t1 vs t4: *p* = 0.030 t1 vs t5: *p* = 0.026
Controls	43.46 ± 4.09	45.40 ± 3.92	46.94 ± 3.66	45.81 ± 3.22	47.12 ± 2.58	
*p*	0.013	0.017	0.028	0.002	< 0.001	
Tibialis anterior muscles						
Patients	55.37 ± 1.47	55.35 ± 1.71	56.78 ± 1.41	59.10 ± 1.60	58.62 ± 1.86	t1 vs t4: *p* = 0.024
Controls	51.17 ± 1.58	49.23 ± 1.78	50.95 ± 1.36	52.62 ± 1.66	53.27 ± 1.54	
*p*	ns	0.041	0.018	0.019	0.038	

In the muscles of the extremities, GSVs are higher in the ICU patients within 24 h (biceps brachii and rectus femoris muscles) to 72 h (tibialis anterior muscles) after admission to the ICU. Time after admission to the ICU: t1: < 24 h; t2: 3 days; t3: 5 days; t4: 8 days; t5: 16 days

Time course analysis detected significantly higher GSVs within the patient group in the biceps brachii muscles (*F* = 14.761; *p* = 0.001). Post-hoc tests revealed higher GSVs among the patients at all time points (t1: *p* = 0.017; t2: *p* = 0.008; t3: *p* = 0.010; t4: *p* = 0.001; t5: *p* = 0.001, *t* tests). For details, see Fig. 3b.

Time course analysis detected significantly higher GSVs within the patient group in the rectus femoris muscles (*F* = 9.455; *p* = 0.005). Post-hoc tests revealed higher GSVs among the patients at all time points (t1: *p* = 0.013; t2: *p*  = 0.017; t3: *p* = 0.028; t4: *p* = 0.002; t5: *p* < 0.001, *t* tests). Among the patients, the GSVs further increased compared with t1 at t4 (*p* = 0.030) and t5 (*p* = 0.026). For details, see Fig. 3c.

Time course analysis detected significantly higher GSVs within the patient group in the tibialis anterior muscles (*F* = 7.282; *p* = 0.012). Post-hoc tests revealed higher GSVs among the patients at t2–t5 (t2: *p* = 0.041; t3: *p* = 0.018; t4: *p* = 0.019; t5: *p* = 0.038, *t* tests). Among the patients, the GSVs further increased compared with t1 at t4 (*p* = 0.024). For details, see Fig. 3d.

No fluctuations in the GSVs within the healthy control group were seen. GSVs within the control group were lower compared with the patient group in all muscles at all time points.

### Nerve sonography

**Fig. 4:**
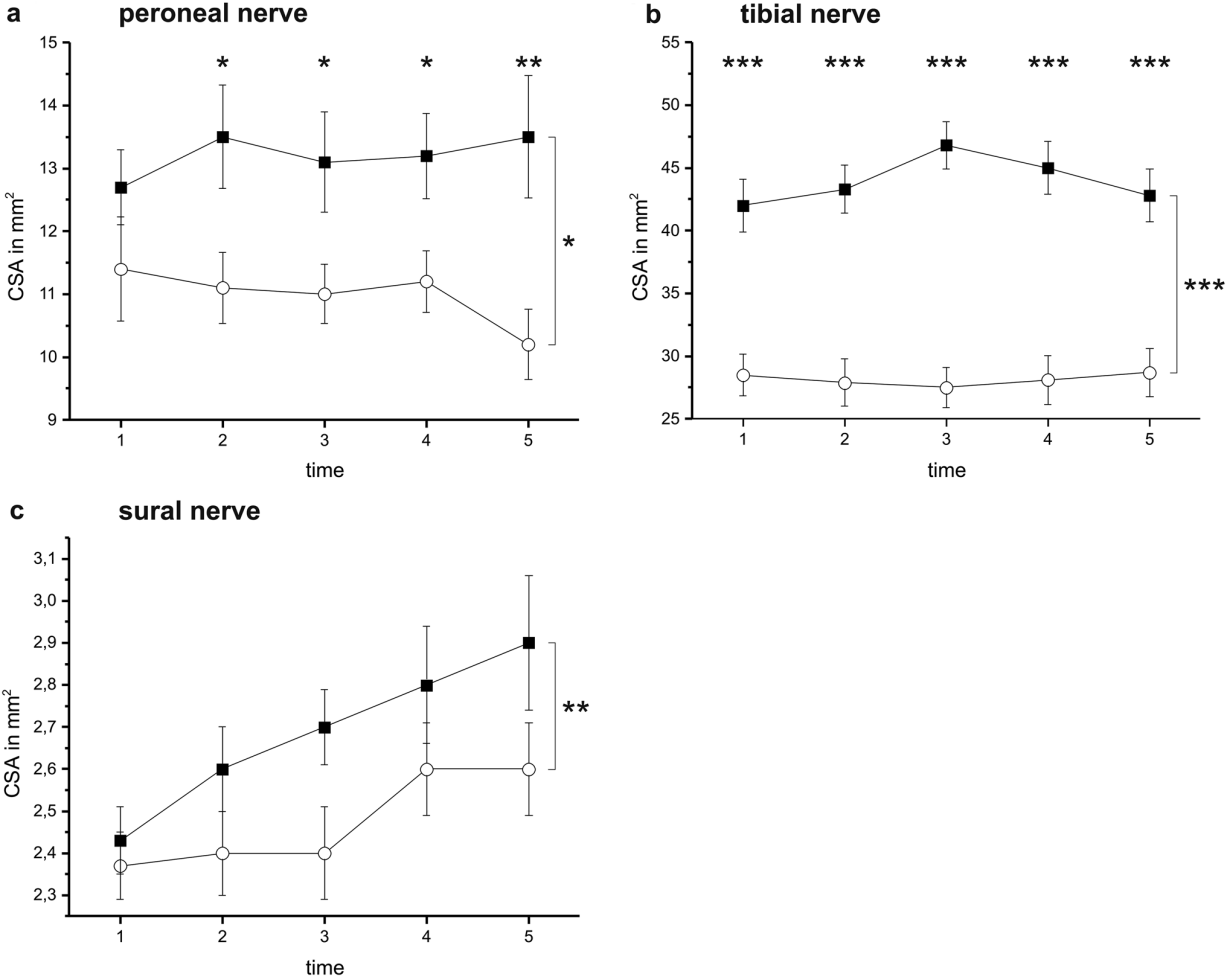
Depicts the time course of the cross-sectional area (CSA) in ICU patients and the healthy control group. The CSA is higher in the patients compared with healthy controls for all of the investigated nerves (**a**: peroneal nerve: *F* = 7.129; *p* = 0.014; **b**: tibial nerve: *F* = 28.976, *p* < 0.001; **c**: sural nerve: *F* = 13.051; *p* = 0.001). Statistical details are presented in Table 2. ***p < 0.001; **p < 0.01; *p < 0.05.

Time course analysis detected a significantly larger CSA of the peroneal nerve within the patient group (*F* = 7.129; *p* = 0.014). Post-hoc tests revealed a significantly larger CSA within the patient group at t2 (*p* = 0.019), t3 (*p* = 0.032), t4 (*p* = 0.026), and t5 (*p* = 0.008; *t* tests). For details, see Fig. 4a and Table 3.

**Table 3:** CSA of nerves

Nerves	CSA in mm^2^					
	t1	t2	t3	t4	t5	
Peroneal nerves						
Patients	12.7 ± 0.60	13.5 ± 0.82	13.1 ± 0.80	13.2 ± 0.68	13.5 ± 0.97	
Controls	11.4 ± 0.83	11.1 ± 0.56	11.0 ± 0.47	11.2 ± 0.49	10.2 ± 0.56	
*p*	0.218	0.019	0.032	0.026	0.008	
Tibial nerves						
Patients	42.0 ± 2.12	43.3 ± 1.92	46.8 ± 1.90	45.0 ± 2.12	42.8 ± 2.09	t1 vs t3: *p* = 0.013 t1 vs t4: *p* = 0.002
Controls	28.5 ± 1.68	27.9 ± 1.88	27.5 ± 1.58	28.1 ± 1.93	28.7 ± 1.94	
*p*	< 0.001	< 0.001	< 0.001	< 0.001	< 0.001	
Sural nerves						
Patients	2.4 ± 0.08	2.6 ± 0.10	2.7 ± 0.09	2.8 ± 0.14	2.9 ± 0.16	t1 vs t2: *p* = 0.033; t1 vs t3: *p* = 0.015; t1 vs t4 *p* = 0.007; t1 vs t5: *p* = 0.010
Controls	2.4 ± 0.08	2.4 ± 0.10	2.4 ± 0.11	2.6 ± 0.11	2.6 ± 0.11	
*p*	0.707	0.175	0.137	0.320	0.155	

The CSA of the motor nerves is enlarged in the patient group after 24 h (tibial nerve) or 72 h (peroneal nerves) after ICU admission. The CSA of the tibial nerves and the sural nerves further increases during the ICU stay. The conventions are as in Table 1

Time course analysis detected a significantly larger CSA of the tibial nerve within the patient group (*F* = 28.976; *p* < 0.001). Post-hoc tests revealed a significantly larger CSA within the patient group at all time points of our investigation (t1, t2, t3, t4, and t5: all *p* < 0.001; *t* tests). Among the patients, the CSA further increased at t3 (*p* = 0.013) and t4 (*p* = 0.002) compared with the baseline. For details, see Fig. 4b.

Time course analysis detected a significantly larger CSA of the sural nerve within the patient group (*F* = 13.051; *p* = 0.001). Post-hoc tests did not reveal significant differences between the investigated groups (ns). Among the patients, the CSA further increased at all observation points (t2: *p* = 0.033; t3: *p* = 0.015; t4 *p* = 0.007; t5: *p* = 0.010) compared with the baseline. For details, see Fig. 4c.

### CMAP

The CMAP of the tibial nerve did not differ between the patients (t1: 10.077 ± 0.89 mV) and healthy participants (t1: 11.475 ± 1.80 mV; ns). The CMAP remained stable in the patients during our observation period (CMAP in mV: t2: 9.704 ± 0.92; t3: 9.704 ± 0.99; t4: 9.814 ± 0.83; t5: 9.973 ± 0.80; ns).

### Correlations

No relevant correlation between the laboratory tests and the GSVs as well as the CSA could be detected. No relevant correlations between GSVs and clinical outcome data could be established. Positive correlations were detected between the CSA of the tibial nerve and the ventilation time (t1: *p* = 0.003; *r* = 0.499; t2: *p* = 0.030; *r* = 0.373; t3: *p* = 0.003; *r* = 0.516; t4: *p* < 0.001; *r* = 0.845) and the duration of the stay in the ICU (t1: *p* = 0.001; *r* = 0.533; t2: *p* = 0.015; *r* = 0.414; t3: *p* = 0.001; *r* = 0.551; t4: *p* < 0.001; *r* = 0.855). Positive correlations were detected between the CSA of the peroneal nerve and the ventilation time (t2: *p* = 0.033; *r* = 0.377; t3: *p* = 0.009; *r* = 0.467; t4: *p* < 0.001; *r* = 0.680) and the duration of the patient’s stay in the ICU (t2: *p* = 0.017; *r* = 0.420; t3: *p* = 0.015; *r* = 0.440; t4: *p* < 0.001; *r* = 0.688).

## Discussion

We provide a longitudinal study with five repetitive measurements of quantitative muscle sonography between days 1 and 16 after admission to the ICU using gray-scale analysis and nerve ultrasound in comparison with age- and sex-matched healthy volunteers. In ICU patients, muscle echogenicity and the nerve CSA increased in the patient group, with the occurrence of these changes taking place very early (24–72 h) during the ICU stay. The CSA of the motor nerves showed an association with the ventilation time and duration of the ICU stay. Our data indicated that neuromuscular changes are accessible by sonography, occur very early during an ICU stay, and even precede electroneurographical changes.

The diagnostic accuracy of muscle ultrasound with a sensitivity of 82% and a specificity of 57% for diagnosing probable ICU-AW appears to be moderate [7]. However, this previous study used the semiquantitative Heckmatt-scale [18] to detect changes in muscle echogenicity. The advantage of computer-assisted gray-scale analysis is its higher sensitivity for detecting neuromuscular diseases in children, which renders analysis of GSVs a more objective method compared with the Heckmatt scale [19]. A previous study using quantitative muscle sonography found increased muscle echogenicity after 1 and 2 weeks compared with the day of admission in the ICU in the tibialis anterior and the rectus femoris but not in the biceps brachii muscles [20] in traumatic brain injury patients. Moreover, it has been shown that the echogenicity of the rectus femoris and vastus intermedius muscles increases over a 10-day ICU period [21]. However, in the mentioned studies, the muscle echogenicity was not compared with a healthy control group. Grimm and co-workers observed an increase in the echotexture at days 4 and 14 in patients with severe sepsis or septic shock after the onset of sepsis compared with healthy controls using the Heckmatt-scale [22]. The prevalence of CIM in sepsis is very high (approximately 70%) and with additional multiple organ failure even almost 100% [23]. None of our patients developed sepsis or multiple organ failure. Due to our study protocol with GSV analysis and a healthy control group, we were already able to detect ICU-induced changes within 24 h of admission, as well as a further increase in echogenicity in the muscles of the lower extremities. These monitored changes can be interpreted as a decrease in muscle quality, which can be relevant for rehabilitation. A strong correlation among changes in echogenicity—for example, muscle quality, strength, and functional outcome—was previously described for the muscles in the thigh [21]. Moreover, increased muscle echogenicity was associated with a reduced likelihood of discharge as well as increased mortality [7]. However, upon awakening, quantitative changes in muscle sonography cannot discriminate between patients with and without ICU-AW [9]. Repetitive measurements might be necessary for identifying changes in echogenicity to correctly identify patients at risk of ICU-AW as performed in the present study. Our results with very early elevated echogenicity in all muscles, and the further increase of echogenicity in the tibialis anterior as well as the rectus femoris muscles indicate that the muscles in the lower extremity might specifically be susceptible to ICU-induced changes. Muscle sonography is feasible for detecting changes in muscle quality very early during an ICU stay, which might have implications for the clinical outcomes.

Muscle edema leads to slightly decreased echogenicity on a sonograph [24]. Zaidman and colleagues state that sonography is unable to detect an edema-like pathology in early or mild inflammatory myopathies [25]. Therefore, our very-early-detected increase in muscle echogenicity cannot simply be attributed to confounding factors, such as excessive fluid administration, which often present in the first days after ICU admission. Moreover, echogenicity further increased within our observation period in the muscles of the lower extremity. During an ICU stay, the first histological changes in muscles with facial inflammation are visible at day 1 [26]. Accompanying myofiber necrosis occurs later and predicts increased ultrasound echogenicity during the first week of critical illness [26]. At day seven, 50% of the patients already showed muscle necrosis [4]. In CIM, muscle biopsies showed the loss of thick filaments, such as myosin and varying degrees of necrosis [27, 28]. Therefore, our results underpin that pathological changes within the muscles occur very early during the ICU stay. However, obviously, not all patients develop ICU-AW. Our results prepare the road for further studies investigating echogenicity changes with clinical outcome parameters.

No studies have prospectively assessed the evolution of CIP starting at the time of ICU admission in patients [2, 29]. In severe sepsis, CIP-induced electroneurographic changes occur 4 days after admission [22]. In our patient group, CMAP stayed unchanged within 16 days of observation. The exact mechanisms of axonal injury in CIP are still unknown. It is hypothesized that nerve damage derives from increased vascular permeability, causing endoneurial edema and subsequent hypoxia [30]. In one previous study, the diagnostic accuracy of CSA of the median and the peroneal nerve was low for diagnosing ICU-AW at the time of awakening [9]. Although CIP is known to be an axonal neuropathy associated only with mild CSA enlargement [31], we observed an enlarged CSA in the peroneal and tibial nerve very early during the ICU stay compared with healthy controls and a further increase in the size of the tibial and sural nerves over time. These changes precede CMAP changes. Interestingly, the CSA of the tibial and peroneal nerves showed positive associations with the ventilation time and the duration of the ICU stay. This aspect is relevant because CIP is associated with a failure to wean from the ventilator [2], and nerve ultrasound might be able to identify patients at risk of developing ICU-AW. We hypothesize that CIP-induced changes are accessible via ultrasound and that the true time of CIP onset is very early during the ICU stay. Because Witteveen and colleagues [9] did not detect differences between ICU patients and controls, our findings cannot be interpreted as results from confounding factors, such as generalized edema. The tibial nerve might especially be susceptible because the nerve enlargement is already obvious within 24 h after ICU admission, and the size further increases. Further studies are needed to understand the association between ultrasound findings and clinical outcomes. Moreover, sonographic examinations directly after admission to the ICU would be interesting for detecting the exact time of origin of the detected changes.

The major limitation of our study is that we investigated patients that presented with paralyses due to cerebral pathologies. Therefore, clinical outcome parameters were difficult to define, and no systematic follow-up of the patients was carried out after the study protocol was completed. We investigated only patients who were ventilated during the entire observation period. Therefore, a clinical examination was not possible. However, our findings regarding the increased muscle echogenicity and the increased CSA are valid but need to be put into a clinical context in future studies.

## Conclusion

Our findings regarding increased echogenicity and an enlarged CSA just 24–72 h after ICU admission point to very early changes in muscles and nerves that might be associated with the development of ICU-AW. Especially the correlation between CSA enlargement and ventilation time deserves further investigation. Therefore our observations might be severity signs of neuromuscular suffering for the most severe patients.

## Declarations

### Availability of data and material

The data can be obtained from the corresponding author upon reasonable request.

### Code availability

N.A.
